# Increased Serum Sclerostin Level Is a Risk Factor for Peripheral Artery Disease in Patients with Hypertension

**DOI:** 10.3390/medicina61071204

**Published:** 2025-07-01

**Authors:** Yahn-Bor Chern, Po-Sheng Lee, Ji-Hung Wang, Jen-Pi Tsai, Bang-Gee Hsu

**Affiliations:** 1 Division of Nephrology, Department of Internal Medicine, Yuan’s General Hospital, Kaohsiung 80249, Taiwan; 2 Department of Internal Medicine, Hualien Tzu Chi Hospital, Buddhist Tzu Chi Medical Foundation, Hualien 97004, Taiwan; 3 Division of Cardiology, Hualien Tzu Chi Hospital, Buddhist Tzu Chi Medical Foundation, Hualien 97004, Taiwan; 4 School of Medicine, Tzu Chi University, Hualien 97004, Taiwan; 5 Division of Nephrology, Department of Internal Medicine, Dalin Tzu Chi Hospital, Buddhist Tzu Chi Medical Foundation, Chiayi 62247, Taiwan; 6 Division of Nephrology, Hualien Tzu Chi Hospital, Buddhist Tzu Chi Medical Foundation, Hualien 97004, Taiwan

**Keywords:** sclerostin, dickkopf-1, hypertension, peripheral artery disease, ankle-brachial index

## Abstract

*Background and Objectives*: Sclerostin and dickkopf-1 (DKK1), which are Wnt inhibitors, are involved in vascular calcification and atherosclerosis. Atherosclerotic peripheral artery disease (PAD) is highly prevalent, particularly in patients with hypertension. This study aimed to explore the association between serum concentrations of Wnt pathway inhibitors and PAD in patients with hypertension. *Materials and Methods*: This cross-sectional trial recruited 92 patients with hypertension. PAD was defined as an ankle-brachial index < 0.9. The levels of sclerostin, DKK1, C-reactive protein (CRP), and other biochemical markers were assessed using fasting blood samples. Univariate and multivariate logistic regression and receiver operating characteristic curve analyses were conducted. *Results*: Patients with PAD (15.2%) had significantly higher serum sclerostin (*p* < 0.001) and CRP (*p* = 0.001) levels than those without PAD. However, the two groups did not significantly differ in terms of the DKK1 levels. Based on the multivariate analysis, sclerostin was an independent predictor of PAD (odds ratio: 1.054 per 1 pmol/L increase, 95% confidence interval: 1.019–1.090, *p* = 0.002) after adjusting for body mass index, fasting glucose levels, diabetes, smoking, and CRP levels. Sclerostin had a strong discriminatory power for diagnosing PAD according to the receiver operating characteristic curve analysis (area under the curve: 0.806, *p* < 0.001), with the best cutoff value of 71.5 pmol/L (sensitivity: 71.4%, specificity: 78.2%). Further, sclerostin was negatively associated with the ankle-brachial index, renal function, and dyslipidemia markers. *Conclusions*: Serum sclerostin levels are independently related to an increased risk for PAD in patients with hypertension. Therefore, it can be a potential biomarker for risk stratification and early diagnosis.

## 1. Introduction

Hypertension remains the most prevalent modifiable risk factor of atherosclerotic cardiovascular disease (ASCVD). However, its control rates have plateaued or even declined in several populations despite guideline-directed therapies [1,2,3,4]. Recent U.S. data have shown that less than one-quarter of adults with hypertension achieve a target blood pressure (BP) below 130/80 mmHg. Moreover, hypertension control has not improved over the last 2 years despite increased treatment efforts [3]. Peripheral arterial disease (PAD) is the manifestation of atherosclerosis in the lower limbs. Its global prevalence varies considerably. In particular, it ranges from approximately 5% in general adult populations to >20% among older age groups, and it is even higher in individuals with hypertension [5,6,7]. In addition to claudication and limb-threatening ischemia, PAD is associated with a 3- to 5-fold higher risk of myocardial infarction and stroke, thereby underscoring its role both as a marker and amplifier of systemic vascular risk [8,9,10].

Atherosclerosis is a multifactorial disease process that involves endothelial injury, inflammation, oxidative stress, lipid deposition, vascular smooth muscle cell (VSMC) proliferation, and plaque formation, which results in arterial narrowing. Wnt signaling is an important pathway that is upregulated in atherosclerosis and is associated with nearly all pathogenic processes (i.e., endothelial injury, inflammation, VSMC activity, plaque formation, and calcification) at the molecular level [11,12,13,14]. There are three downstream signalings of the Wnt pathway, and the canonical Wnt/β-catenin pathway is the most relevant to atherosclerosis [13]. Two endogenous Wnt antagonists, sclerostin and dickkopf-1 (DKK1), interrupt signaling transduction by binding to the coreceptors low-density lipoprotein receptor-related protein 5/6, in association with the Wnt protein/Frizzled receptor. Thus, they are two of the most important Wnt inhibitory factors in vivo [15,16]. The circulating levels of Wnt inhibitors mirror vascular disease burden and the activity of vascular disease. Based on previous studies, higher serum sclerostin or DKK1 levels are independently associated with coronary artery disease, stroke, PAD, and carotid atherosclerosis [17,18,19,20,21]. Hence, these factors may be biomarkers of subclinical or overt cardiovascular disease. The current study aimed to explore the association of serum sclerostin or DKK1 with PAD in patients with hypertension. We hypothesized that higher levels of Wnt inhibitory factors are associated with an increased prevalence of PAD measured using the ankle-brachial index (ABI) in this high-risk cohort. If validated, the results may support the use of circulating sclerostin and DKK1, which can be easily accessed and are noninvasive, as biomarkers for risk stratification in the context of PAD among patients with hypertension. In turn, this leads to the provision of individualized preventive and therapeutic measures.

## 2. Materials and Methods

### 2.1. Participants

This prospective cross-sectional study was conducted between July 2020 and December 2020. In total, 92 adult patients (aged ≥ 18 years old) with hypertension from the cardiovascular outpatient clinics of a medical center in Hualien, Taiwan, were enrolled. Patients with an ABI > 1.3, those with a recent history of acute myocardial infarction or acute decompensated heart failure within 2 months, malignancy, chronic inflammatory conditions (such as inflammatory bowel disease and rheumatoid arthritis), and acute infectious diseases at baseline blood sampling, or those who refused to provide the written informed consent to participate in the study were excluded from the analysis. The Research Ethics Committee of Hualien Tzu Chi Hospital, Buddhist Tzu Chi Medical Foundation (IRB108-219-A), approved this study. Written informed consent was obtained from all the participants in this study. For BP measurement, our participants rested for at least 10 min, and their morning BP was measured by the trained staff using standard mercury sphygmomanometers with the appropriate cuff size. The systolic BP (SBP) and diastolic BP (DBP) were determined at the onset and disappearance of the Korotkoff sounds, respectively. BP was recorded three times at 5 min intervals by well-experienced and trained staff members, and their means were used in the analysis. In the prevalence survey, hypertension was defined as an SBP ≥ 140 mmHg and/or a DBP ≥ 90 mmHg, or the intake of antihypertensive medications within the last 2 weeks, as per the Eighth Report of the Joint National Committee on Prevention, Detection, Evaluation, and Treatment of High Blood Pressure criteria. Diabetes mellitus (DM) was defined as a fasting plasma glucose level ≥126 mg/dL, post-prandial glycemic levels ≥ 200 mg/dL with symptoms related to glucose toxicity (or two instances of post-prandial glycemic levels ≥ 200 mg/dL in asymptomatic patients), hemoglobin A1c level ≥ 6.5%, or the use of antidiabetic agents.

### 2.2. Anthropometric Evaluation

The patients were examined in the morning and after an overnight (at least 8 h) fast. Their body weight and height were recorded to the nearest 0.5 kg and 0.5 cm, respectively. Body mass index (BMI) was calculated as weight (kg) divided by height in meters squared (m^2^) [22,23].

### 2.3. Biochemical Analyses

Blood samples were collected under fasting conditions and immediately after centrifugation at 3000 g for 10 min. Then, the abovementioned centrifuged sample was used to measure the serum concentrations of blood urea nitrogen (BUN), creatinine, fasting glucose, total cholesterol, triglyceride, high-density lipoprotein cholesterol (HDL-C), low-density lipoprotein cholesterol (LDL-C), total calcium, phosphorus, and C-reactive protein (CRP) levels with an autoanalyzer (Siemens Advia 1800; Siemens Healthcare GmbH, Henkestr, Germany) [22,23]. The serum human intact parathyroid hormone levels (Abcam, Cambridge, MA, USA) and the serum sclerostin and serum DKK1 levels (Biomedica Immunoassays, Vienna, Austria) were measured using commercially available enzyme-linked immunosorbent assays [24,25]. The estimated glomerular filtration rate (eGFR) of each patient was calculated using the chronic kidney disease epidemiology collaboration equation.

### 2.4. ABI Measurements

BP was measured on three occasions using the oscillometric method in the upper and lower extremities while the patients were in the supine position and in the brachial, dorsalis pedis, and posterior tibial arteries (VaSera VS-1000; Fukuda Denshi, Tokyo, Japan) [26,27]. The ABI for the right or left side was defined as the ratio of the highest recorded SBP in the respective ankle (either the dorsalis pedis or the posterior tibial artery) to the highest recorded SBP in the brachial artery of either arm. Continuous electrocardiographic monitoring was conducted for a duration of >15 min. Patients with a low ABI (<0.9 in either lower extremity) were clinically diagnosed with PAD, as mentioned in previous studies [26,27].

### 2.5. Statistical Analysis

A power analysis was conducted, which showed that the study was powered to detect a correlation between sclerostin and ABI [28]. The power analysis was based on a moderate effect size (*r* = 0.30) at α = 0.05 and β = 0.20 (equivalent to power = 80%). The sample size estimate showed that at least 85 patients must be included to achieve statistical power to detect an association of this size. The last population recruited included 92 patients with hypertension, which surpassed the estimated sample size and, therefore, ensured that the study was properly powered to detect the predicted correlation.

The normality of the data was tested with the Kolmogorov–Smirnov test. Continuous data were presented as the means and standard deviation in variables with a normal distribution and as the medians and interquartile ranges (IQRs) in variables with a non-normal distribution for between-group comparisons (two-tailed independent Student’s *t*-test and the Mann–Whitney U test, respectively). Categorical variables were presented as numbers with percentages and analyzed using the χ2 test. Variables that had a *p*-value < 0.2, such as sclerostin levels, fasting glycemic levels, BMI, CRP levels, smoking status, and the presence of DM, were considered as the potential risk factors of PAD in the univariate analysis. These variables were further subjected to multivariable logistic regression analysis. The area under the curve (AUC) and the serum sclerostin level for the prediction of PAD in patients with hypertension were estimated using the receiver operating characteristic curve. The nonparametric Spearman’s rank correlation coefficient was used to determine the correlation of left ABI, right ABI, and sclerostin concentrations with clinical parameters. The Statistical Package for the Social Sciences software (version 19.0, IBM Corp., Armonk, NY, USA) on Windows was used for the statistical analysis. A *p*-value < 0.05 was considered statistically significant.

## 3. Results

Table 1 shows the demographic and clinical characteristics and laboratory findings of the 92 patients with hypertension, including 14 (15.2%) in the low-ABI (PAD) group and 78 (84.8%) in the normal-ABI group. The low-ABI group had a significantly higher prevalence of DM (57.1% vs. 29.5%; *p* = 0.044) and elevated median CRP levels (0.26 [IQR: 0.23–1.16] vs. 0.20 [IQR: 0.15–0.24] mg/dL; *p* = 0.001) and smoking (*p* = 0.037) compared with the normal-ABI group. The low-ABI group was more likely to have a higher BMI than the normal-ABI group (28.42 ± 3.67 vs. 26.37 ± 3.79 kg/m^2^; *p* = 0.065). Further, fasting glucose levels were found to be related to DM. Thus, both BMI and fasting glucose levels were included as potential confounders of the possible predictors of PAD in the multivariate models. The low-ABI group had a significantly higher mean serum sclerostin level than the normal-ABI group (83.23 ± 23.40 vs. 54.59 ± 22.85 pmol/L; *p* < 0.001). Meanwhile, the circulating DKK1 levels did not differ significantly between the two groups (*p* = 0.639). Moreover, there were no significant differences between the two groups in terms of age, sex, BP, lipid profile, renal function, or the use of antihypertensive, antiplatelet, and statin medications (all *p* > 0.05).

The adjusted odds ratio (OR) for PAD was obtained via multivariate logistic regression analysis (Table 2) of the variables with the *p*-value < 0.2, as shown in Table 1. After adjusting for BMI, fasting glucose levels, DM, smoking, and CRP levels, every 1 pmol/L increase in the serum sclerostin level was related to a 5.4% increase in the odds of having PAD, on average (OR: 1.054, 95% confidence interval [CI]: 1.019–1.090; *p* = 0.002). None of the other covariates in this model were statistically significant.

**Figure 1:**
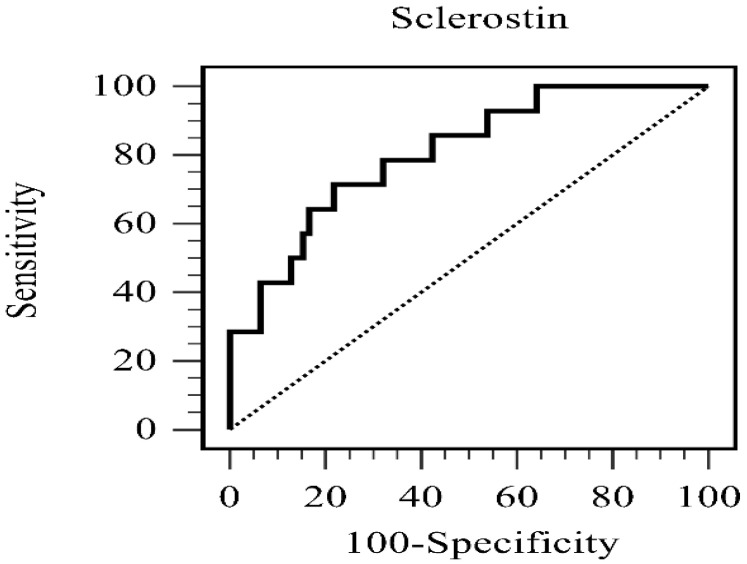
The area under the receiver operating characteristic curve signifies the diagnostic efficacy of sclerostin concentration in forecasting peripheral arterial disease among a cohort of 92 individuals diagnosed with hypertension.

Figure 1 shows the ROC curve for serum sclerostin levels in distinguishing hypertensive patients with PAD from those without. The AUC was 0.806 (95% CI: 0.710–0.881; *p* < 0.001), which indicated good overall discrimination, well above the chance discrimination level of 0.5. At the best cutoff value of 71.5 pmol/L, the sensitivity and specificity were 71.4% and 78.2%, respectively, with a positive likelihood ratio of 3.28 and a negative likelihood ratio of 0.37. Based on these findings, a cutoff serum sclerostin level higher than the described threshold could indicate a 3-fold higher risk of developing PAD. Meanwhile, a level lower than this one represented a significantly lower risk of developing the disease. Therefore, serum sclerostin has a clinically important predictive value for PAD in this population with hypertension.

In the correlation analyses (Table 3), left ABI was strongly associated with right ABI (*r* = 0.617, *p* < 0.001). Further, it was significantly inversely associated with serum sclerostin (*r* = −0.251, *p* < 0.001), LDL-C (*r* = −0.212, *p* =0.043), and log-transformed CRP (*r* = −0.222, *p* = 0.033) levels. Similarly, right ABI was positively correlated with left ABI (*r* = 0.617, *p* < 0.001) and was negatively correlated with sclerostin (*r* = −0.371, *p* < 0.001) and log-CRP (*r* = −0.357, *p* < 0.001) levels. A significant association between ABI and other variables was not observed. In addition to ABI, serum sclerostin levels were significantly correlated with several risk factors. In particular, the sclerostin levels increased with higher BMI (*r* = 0.231, *p* = 0.027), BUN levels (*r* = 0.217, *p* = 0.038), and creatinine levels (*r* = 0.275, *p* = 0.008). The sclerostin levels decreased with a higher eGFR (*r* = −0.222, *p* = 0.033). Further, an increasing level of sclerostin was related to a low serum HDL-C level (*r* = −0.210, *p* = 0.044) and a low serum phosphorus level (*r* = −0.253, *p* = 0.015). Based on these results, sclerostin is an index of not only peripheral arterial perfusion but also general metabolic and kidney-related conditions.

## 4. Discussion

The current study showed that compared with the patients without PAD, those with PAD had a higher diabetes prevalence, smoking, and CRP levels. Further, they tended to have a higher BMI, which was also considered as a potential confounder in the multivariate models, along with the fasting glycemic assay (since it is associated with DM). Of interest, the patients with PAD had a significantly higher mean serum sclerostin level than the controls. However, this was not true for DKK1 levels. After adjusting for BMI, fasting glucose levels, diabetes, smoking, and CRP levels, independent of other cardiovascular risk factors, every 1 pmol/L increase in sclerostin levels was associated with a 5.4% increase in the risk of developing PAD. Further, the ROC analysis showed a good discriminative ability (AUC: 0.806). The negative association between sclerostin levels and left and right ABI was confirmed via correlation analyses. In addition, higher sclerostin levels were associated with increased BMI, BUN levels, and creatinine levels and a lower eGFR, HDL-C level, and phosphorus level. These findings suggest that sclerostin reflects not only peripheral arterial perfusion but also broader aspects of metabolic and renal health.

DM and CRP are well-established mechanistic factors in PAD. From a mechanistic perspective, DM can lead to PAD via different mechanisms, such as impaired endothelial function, accelerated atherosclerosis secondary to hyperglycemia and insulin resistance, and oxidative stress, all of which can deteriorate the vasculature and result in arterial stenosis [29,30,31]. In addition, CRP, a marker of systemic inflammation and an acute-phase reactant, is also related to various inflammatory cascades and implicated in atherogenesis [32,33]. This finding was also supported by our initial univariate analysis, which showed a significantly higher frequency of DM and increased median CRP levels in individuals with a low ABI. However, DM and CRP levels were not independent factors associated with PAD based on the following multivariate logistic regression analysis. This lack of multivariate significance is noteworthy as these effects may be mediated by other variables in the models (i.e., the highly predictive sclerostin) or could indicate a relatively low statistical power in this cohort to detect more subtle independent effects when adjusting for other variables. The log-transformed CRP was considered insignificant in the multivariate model for PAD status. However, it was significantly inversely associated with the left and right ABIs in the Spearman correlation analysis. This finding emphasizes an inverse association. In particular, increased systemic inflammation (as indicated by CRP levels) and decreased ABI values correspond to a more severe PAD. Statistical discrepancies between this apparent bivariate relationship and the multivariate findings indicate that the relationship between inflammation and PAD is robust. Further, the independent predictive potential of CRP for the presence of PAD might be somehow reduced if other covariates that could be either confounders or intermediaries (i.e., DM and sclerostin) are simultaneously accounted for. The multivariate analysis controlled for these confounding relations and provided an image of the independent contribution. However, the correlation analysis only showed the primary association.

Sclerostin is a protein that is mainly secreted by osteocytes in the bone matrix. Further, it is a potent inhibitor of bone formation, mainly by inhibiting the Wnt pathway [13]. However, in addition to its well-defined function in regulating the skeleton, a growing body of evidence suggests that sclerostin plays a role in extra-skeletal processes, particularly vascular pathologies [13]. Our study adds to this growing knowledge because the correlation analysis not only corroborated its association with ABI but also showed the association of serum sclerostin with various metabolic and renal markers. The association between bone and adiposity was confirmed by the correlation between sclerostin and BMI. It is most likely attributable to the following: First, adipose tissue itself can secrete factors (i.e., TNF-α or reactive oxygen species) which may affect osteocyte activity and sclerostin production. Second, insulin resistance or mechanical loading (which is related to a higher BMI) affects sclerostin levels [34,35,36,37]. Further, the strong associations between renal function markers—specifically, positive correlations with BUN and creatinine levels and a negative correlation with eGFR—are particularly noteworthy. This pattern strongly indicates that declining renal function is associated with increased circulating sclerostin, likely due to impaired renal clearance of sclerostin, increased production of sclerostin by osteocytes in response to renal injury as a compensatory or self-defense mechanism, interaction with other chronic kidney disease biomarkers (such as fibroblast growth factor-23), and activation of the Wnt/β-catenin signaling or activin (transforming growth factor-β family) [38,39,40,41,42]. In addition, the negative correlation between sclerostin and HDL-C levels points toward a connection with dyslipidemia. A lower HDL-C level is a common cardiovascular risk factor, and elevated sclerostin levels might contribute to or be a marker of an atherogenic endothelial environment [43,44]. Finally, the inverse association of sclerostin with serum phosphorus is an interesting finding. Although hyperphosphatemia is frequently reported to be associated with vascular issues in patients with renal conditions, the negative association observed in the hypertensive cohort may similarly indicate complex regulatory mechanisms between bone turnover and mineral metabolism. However, this should be further investigated [45,46]. Taken together, these associations indicate that sclerostin is a biomarker of not only PAD but also more general systemic metabolic and renal health issues. Sclerostin levels are elevated in both type 1 and type 2 diabetes due to a combination of metabolic and vascular factors [47,48]. As noted before, sclerostin is associated with insulin resistance and rises significantly with declining renal function and may play a protective or regulatory role in vascular and mineral metabolism [34,35,36,37,38,39,40,41,42]. In our study, no significant association was observed between serum sclerostin levels and log-glucose levels. This discrepancy may be due to differences in study population, sample size, or confounding factors such as medication use, glycemic control status, or comorbidities. Further studies with larger cohorts and comprehensive metabolic profiling are needed to clarify the relationship between sclerostin and glucose metabolism.

The implication of this study is that sclerostin is both a marker and mediator of PAD measured using ABI in individuals with hypertension. More importantly, the participants with a low ABI had significantly higher mean sclerostin levels. This finding was further confirmed by performing multivariate logistic regression analysis, which showed that sclerostin is an independent predictor of PAD. The clinical relevance of sclerostin was also underscored by its good discriminatory power for PAD in the ROC curve analysis. The Spearman correlation analysis confirmed a significant inverse association between sclerostin levels and ABI, thereby indicating that higher sclerostin levels are associated with a more impaired peripheral perfusion. This strong association can likely be explained by several pathogenic mechanisms. First, an inappropriate activation of the Wnt signaling has been observed in calcified atherosclerotic and aneurysmal lesions [49]. Then, a high sclerostin level is attributed to increased signaling of the Wnt pathway in atherosclerotic lesions involving mechanical unloading and vascular calcification. This is because the shearing force is an important trigger responsible for the upregulation of the Wnt pathway [50]. Thereafter, endothelial dysfunction, development of pro-inflammatory status, monocyte migration, foam cell formation, smooth muscle cell proliferation, and eventual osteoblast transition into the arterial walls are all implicated in this process [51,52,53,54,55]. Therefore, elevated sclerostin levels in patients with PAD could be a part of a self-defense mechanism in response to the overall inappropriate activation of the Wnt pathway. These findings suggest that sclerostin is an important molecule in the “bone-vascular axis”, in which the perturbation of bone-derived factors adversely influences vascular health, thereby contributing to the evolution of PAD. From a clinical standpoint, these data indicate the clinical value of serum sclerostin as a screening biomarker of hypertension in patients at higher risk of developing PAD and those who may benefit from a timely diagnosis or a more aggressive preventive intervention. Further, its putative pathogenic role also introduces an interesting concept showing that sclerostin might be a novel therapeutic target for preventing or treating PAD. Although the correlation analysis between serum sclerostin and clinical parameters such as ABI showed a relatively weak to moderate correlation, this may reflect the multifactorial nature of PAD, in which no single biomarker fully explains disease variance. While the observed R values were statistically significant, they should be interpreted with caution, and further research must be conducted to validate this finding.

The current study has several limitations. First, the study cohort was relatively small, and the participants were from a single center, with only 14 PAD events, which may introduce potential bias or lead to overestimation of effect sizes, particularly in subgroup analyses and discrimination metrics. Moreover, the small sample size and low event rate might have resulted in imprecise estimates and limited applicability to the general population. Second, adjustments for key covariates such as BMI, diabetes status, smoking status, fasting glucose levels, and CRP levels were performed. However, due to the multifactorial nature of atherosclerosis, other potential confounders—such as smoking frequency, duration of hypertension, medication adherence, vascular calcification imaging results, and other inflammatory or metabolic biomarkers—were not identified. Third, this study was dependent on ABI measurements for defining PAD, and it excluded patients with ABI > 1.3. Therefore, our findings cannot be extrapolated to those with extensive vascular calcification. Fourth, the study was cross-sectional in nature. Therefore, a causal inference cannot be drawn. Moreover, residual confounding by unmeasured comorbidities (i.e., peripheral neuropathy and microvascular disease) or lifestyle factors (e.g., physical activity, dietary patterns) might influence both sclerostin levels and the risk of PAD. Clinical variables (e.g., physical activity, claudication symptoms, family history, ankle pressure measurements over time) required to compute validated PAD risk scores, such as the Edinburgh Claudication Questionnaire or Framingham PAD risk model, and vascular imaging modalities to assess the anatomical severity of PAD, were not included in our study. Nevertheless, risk scoring systems, vascular imaging modalities, and long-term clinical outcomes, such as mortality and major adverse cardiovascular events, in larger, prospective cohorts with more diverse populations, and longitudinal designs must be carried out to confirm our findings and to delineate the mechanistic pathways via which sclerostin is related to PAD.

## 5. Conclusions

An independent association was observed between increased serum sclerostin levels and PAD in patients with hypertension. Our findings emphasize that sclerostin is not only significantly elevated in individuals with low ABI but also functions as an independent predictor of PAD, indicating a substantially increased risk. Based on these results, sclerostin can be clinically useful when used as a biomarker for early diagnosis and risk stratification in patients with PAD. Further, it can be a novel therapeutic target. However, the limitations of the current study, which include the small sample size, the low rates of PAD, and the lack of direct data on vascular calcification and potential residual confounders, should be cautiously considered. Subsequent studies involving larger, prospective cohorts must be performed to confirm our findings, and future studies should be carried out to directly examine the role of sclerostin in vascular calcification in PAD pathophysiology and the potential therapeutic value of regulating sclerostin signaling in the prevention or treatment of PAD.

## Figures and Tables

**Table 1:** Clinical characteristics of the 92 hypertensive individuals in the cohort exhibiting either a normal or diminished ankle-brachial index.

Characteristic	All Participants (*n* = 92)	Normal ABI Group (*n* = 78)	Low ABI Group (*n* = 14)	*p* Value
Age (years)	64.76 ± 9.11	64.33 ± 8.71	67.14 ± 11.12	0.290
Height (cm)	160.94 ± 8.22	160.98 ± 8.27	160.71 ± 8.19	0.912
Body weight (kg)	69.37 ± 12.79	68.59 ± 12.65	73.71 ± 13.15	0.169
Body mass index (kg/m^2^)	26.68 ± 3.82	26.37 ± 3.79	28.42 ± 3.67	0.065
Left ankle-brachial index	1.08 ± 0.10	1.10 ± 0.07	0.92 ± 0.11	<0.001 *
Right ankle-brachial index	1.06 ± 0.12	1.09 ± 0.08	0.88 ± 0.14	<0.001 *
Systolic blood pressure (mmHg)	133.02 ± 16.53	132.51 ±17.25	135.86 ± 11.89	0.489
Diastolic blood pressure (mmHg)	74.45 ± 9.71	74.81 ± 9.88	72.43 ± 8.77	0.402
Total cholesterol (mg/dL)	172.98 ± 39.50	171.88 ± 39.56	179.07 ± 40.09	0.534
Triglyceride (mg/dL)	129.00 (98.00–167.00)	124.00 (94.00–176.00)	137.00 (120.00–153.00)	0.700
HDL-C (mg/dL)	44.71 ± 12.76	44.47 ± 12.36	46.00 ± 15.24	0.683
LDL-C (mg/dL)	102.41 ± 31.86	101.14 ± 32.03	109.50 ± 31.05	0.369
Fasting glucose (mg/dL)	110.00 (97.00–144.75)	107.50 (95.75–137.25)	124.00 (101.50–187.50)	0.150
Blood urea nitrogen (mg/dL)	16.96 ± 5.06	16.96 ± 4.44	16.93 ± 7.92	0.982
Creatinine (mg/dL)	1.11 ± 0.31	1.10 ± 0.28	1.21 ± 0.43	0.220
eGFR (mL/min)	67.55 ± 18.96	68.47 ± 18.24	62.47 ± 22.68	0.278
Total calcium (mg/dL)	9.13 ± 0.37	9.13 ± 0.38	9.16 ± 0.33	0.786
Phosphorus (mg/dL)	3.52 ± 0.53	3.53 ± 0.53	3.43 ± 0.54	0.499
Intact parathyroid hormone (pg/mL)	47.70 (32.40–58.53)	45.75 (31.78–58.90)	51.50 (43.33–61.43)	0.325
CRP (mg/dL)	0.21 (0.15–0.28)	0.20 (0.15–0.24)	0.26 (0.23–1.16)	0.001 *
Sclerostin (pmol/L)	58.95 ± 25.04	54.59 ± 22.85	83.23 ± 23.40	<0.001 *
Dickkopf-1 (pmol/L)	16.46 ± 10.79	16.23 ± 11.03	17.71 ± 9.56	0.639
Female, *n* (%)	32 (34.8)	27 (34.6)	5 (35.7)	0.937
Diabetes, *n* (%)	31 (33.7)	23 (29.5)	8 (57.1)	0.044 *
Coronary artery disease, *n* (%)	61 (66.3)	50 (64.1)	11 (78.6)	0.292
Smoking, *n* (%)	14 (15.2)	10 (12.3)	4 (36.4)	0.037 *
ACE inhibitor use, *n* (%)	31 (33.7)	26 (33.3)	5 (35.7)	0.862
ARB use, *n* (%)	53 (57.6)	45 (57.7)	8 (57.1)	0.969
β-blocker use, *n* (%)	54 (58.7)	44 (56.4)	10 (71.4)	0.293
CCB use, *n* (%)	42 (45.7)	37 (47.4)	5 (35.7)	0.418
Statin use, *n* (%)	43 (46.7)	38 (48.7)	5 (35.7)	0.369
Fibrate use, *n* (%)	12 (13.0)	9 (11.5)	3 (21.4)	0.312
Aspirin, *n* (%)	55 (59.8)	46 (59.0)	9 (64.3)	0.709

Values pertaining to continuous variables are expressed as mean ± standard deviation subsequent to evaluation via Student’s *t*-test; variables exhibiting non-normal distribution are represented as median and interquartile range following examination through the Mann–Whitney *U* test; values are delineated as number (%) and subjected to analysis via the chi-square test. (Abbreviations: ABI, ankle-brachial index; HDL-C, high-density lipoprotein cholesterol; LDL-C, low-density lipoprotein cholesterol; eGFR, estimated glomerular filtration rate; CRP, C-reactive protein; ACE, angiotensin-converting enzyme; ARB, angiotensin-receptor blocker; CCB, calcium-channel blocker.) * *p* < 0.05 was considered statistically significant.

**Table 2:** Multivariate logistic regression analysis of the factors correlated with peripheral artery disease among the 92 hypertensive patients.

Variables	Odds Ratio	95% Confidence Interval	*p* Value
Sclerostin, 1 pmoL/L	1.054	1.019–1.090	0.002 *
C-reactive protein, 0.1 mg/dL	1.115	0.964–1.289	0.142
Fasting glucose, 1 mg/dL	0.992	0.967–1.018	0.535
Body mass index, 1 kg/m^2^	1.098	0.899–1.342	0.359
Diabetes mellitus, present	7.792	0.694–87.506	0.096
Smoking, present	6.305	0.791–50.219	0.082

The dataset underwent examination via multivariate logistic regression analysis, incorporating variables such as diabetes mellitus, smoking, body mass index, fasting glucose, C-reactive protein, and sclerostin. * *p* < 0.05 was considered statistically significant.

**Table 3:** Spearman correlation coefficients between left ABI, right ABI, sclerostin, and clinical variables in 92 hypertensive patients.

Variables	Left ABI		Right ABI		Sclerostin (pmol/L)	
	Spearman Coefficient of Correlation	*p* Value	Spearman Coefficient of Correlation	*p* Value	Spearman Coefficient of Correlation	*p* Value
Age (years)	−0.103	0.330	−0.142	0.177	0.204	0.051
Body mass index (kg/m^2^)	−0.080	0.447	0.021	0.840	0.231	0.027 *
Left ABI	—	—	0.617	<0.001 *	−0.251	<0.001 *
Right ABI	0.617	<0.001 *	—	—	−0.371	<0.001 *
Sclerostin (pmol/L)	−0.251	0.016 *	−0.371	<0.001 *	—	—
Dickkopf-1 (pmol/L)	−0.058	0.584	−0.013	0.903	−0.045	0.668
SBP (mmHg)	0.064	0.544	−0.025	0.812	0.039	0.714
DBP (mmHg)	0.134	0.203	0.035	0.739	−0.203	0.052
Total cholesterol (mg/dL)	−0.189	0.071	0.014	0.893	−0.179	0.088
Log-Triglyceride (mg/dL)	0.037	0.723	0.084	0.423	−0.082	0.435
HDL-C (mg/dL)	−0.068	0.518	−0.070	0.505	−0.210	0.044 *
LDL-C (mg/dL)	−0.212	0.043 *	0.017	0.875	−0.077	0.466
Log-Glucose (mg/dL)	−0.002	0.981	0.011	0.917	0.038	0.716
Blood urea nitrogen (mg/dL)	−0.085	0.418	0.033	0.754	0.217	0.038 *
Creatinine (mg/dL)	−0.030	0.774	−0.076	0.471	0.275	0.008 *
eGFR (mL/min)	0.053	0.617	0.060	0.567	−0.222	0.033 *
Total calcium (mg/dL)	0.016	0.882	0.004	0.972	0.001	0.999
Phosphorus (mg/dL)	0.006	0.954	0.020	0.848	−0.253	0.015 *
Log-iPTH (pg/mL)	−0.120	0.254	−0.126	0.230	−0.074	0.483
Log-CRP (mg/dL)	−0.222	0.033 *	−0.357	<0.001 *	0.166	0.114

Data, including triglyceride, glucose, iPTH, and CRP data, were not normally distributed and were thus log-transformed in the analysis. Spearman correlation analysis was performed for data analysis of this correlation study. (Abbreviations: ABI, ankle brachial index; SBP, systolic blood pressure; DBP, diastolic blood pressure; HDL-C, high-density lipoprotein cholesterol; LDL-C, low-density lipoprotein cholesterol; eGFR, estimated glomerular filtration rate; iPTH, intact parathyroid hormone; CRP, C-reactive protein.) * *p* < 0.05 was regarded as statistically significant (two-tailed).

## Data Availability

Upon request, the corresponding author can provide the data utilized in this study.

## Abbreviations

The following abbreviations are used in this manuscript:

DKK1	dickkopf-1
PAD	peripheral artery disease
CRP	C-reactive protein
ASCVD	atherosclerotic cardiovascular disease
VSMC	vascular smooth muscle cell
ABI	ankle-brachial index
BP	blood pressure
SBP	systolic blood pressure
DBP	diastolic blood pressure
DM	diabetes mellitus
BMI	body mass index
BUN	blood urea nitrogen
HDL-C	high-density lipoprotein cholesterol
LDL-C	low-density lipoprotein cholesterol
CRP	C-reactive protein
eGFR	estimated glomerular filtration rate
IQR	interquartile range
AUC	area under the curve
